# Bone Marrow Stromal Cell-Derived IL-8 Upregulates PVR Expression on Multiple Myeloma Cells via NF-kB Transcription Factor

**DOI:** 10.3390/cancers12020440

**Published:** 2020-02-13

**Authors:** Abdelilah Mekhloufi, Andrea Kosta, Helena Stabile, Rosa Molfetta, Alessandra Zingoni, Alessandra Soriani, Marco Cippitelli, Rossella Paolini, Angela Gismondi, Maria Rosaria Ricciardi, Maria Teresa Petrucci, Laura Masuelli, Giulio Caracciolo, Sara Palchetti, Angela Santoni, Cinzia Fionda

**Affiliations:** 1 Department of Molecular Medicine, Istituto Pasteur-Fondazione Cenci Bolognetti, Sapienza University of Rome, 00161 Rome, Italy; mekhloufi.abdelilah@gmail.com (A.M.); andrea.kosta@uniroma1.it (A.K.); helena.stabile@uniroma1.it (H.S.); rosa.molfetta@uniroma1.it (R.M.); alessandra.zingoni@uniroma1.it (A.Z.); alessandra.soriani@uniroma1.it (A.S.); marco.cippitelli@uniroma1.it (M.C.); rossella.paolini@uniroma1.it (R.P.); angela.gismondi@uniroma1.it (A.G.); giulio.caracciolo@uniroma1.it (G.C.); sara.palchetti@uniroma1.it (S.P.); 2 Division of Hematology, Department of Translational Medicine and Precision, Sapienza University of Rome, 00161 Rome, Italy; mariarosaria.ricciardi@uniroma1.it (M.R.R.); petrucci@bce.uniroma1.it (M.T.P.); 3 Department of Experimental Medicine, Sapienza University of Rome, 00161 Rome, Italy; laura.masuelli@uniroma1.it; 4 Neuromed I.R.C.C.S.-Istituto Neurologico Mediterraneo, 86077 Pozzilli, Italy

**Keywords:** PVR, multiple myeloma, natural killer cells, IL-8, bone marrow stromal cells

## Abstract

Bone marrow stromal cells (BMSCs) strongly contribute to multiple myeloma (MM) progression, promoting the survival and growth of malignant plasma cells (PCs). However, the possible impact of these cells on the immune-mediated recognition of MM cells remains largely unknown. DNAM-1 activating receptor plays a prominent role in NK cell anti-MM response engaging the ligands poliovirus receptor (PVR) and nectin-2 on malignant PCs. Here, we analysed the role of MM patient-derived BMSCs in the regulation of PVR expression. We found that BMSCs enhance PVR surface expression on MM cells and promote their NK cell-mediated recognition. PVR upregulation occurs at transcriptional level and involves NF-kB transcription factor activation by BMSC-derived soluble factors. Indeed, overexpression of a dominant-negative mutant of IKBα blocked PVR upregulation. IL-8 plays a prominent role in these mechanisms since blockade of CXCR1/2 receptors as well as depletion of the cytokine via RNA interference prevents the enhancement of PVR expression by BMSC-derived conditioned medium. Interestingly, IL-8 is associated with stromal microvesicles which are also required for PVR upregulation via CXCR1/CXCR2 signaling activation. Our findings identify BMSCs as regulators of NK cell anti-MM response and contribute to define novel molecular pathways involved in the regulation of PVR expression in cancer cells.

## 1. Introduction

Multiple myeloma (MM) is a hematological neoplasia deriving from the uncontrolled proliferation of cancerous plasma cells (PCs) in the bone marrow (BM). Signals from the BM microenvironment and failure of immune surveillance play major roles in the molecular mechanisms driving the progression of this malignancy [1,2,3]. In particular, the complex interaction with bone marrow stromal cells (BMSCs) strongly supports the survival, proliferation and migration of malignant PCs and promotes osteoclastogenesis and angiogenesis [4,5]. BMSC-MM cell communication involves direct adhesive interactions as well as soluble factors able to engage autocrine and paracrine loops, which generate a tumor-promoting microenvironment. BMSC production of growth factors, including IGF-1, HGF and GAS6, or cytokines, such as IL-6 and TNF alpha, and the chemokines SDF-1 and IL-8, sustains MM cell growth and chemoresistance and positively correlates with disease progression [6,7,8]. Most of these soluble factors concur to further induce the activity of NF-kB family transcription factors, which are strongly implicated in the pathogenesis of MM cells [9,10,11]. Moreover, extracellular vesicles (EVs), including microvesicles (MVs) and exosomes, mediate the transport and transfer of various bioactive molecules, such as proteins and RNAs, between MM cells and BMSCs [12,13,14]. In particular, BMSC-derived MVs, which are generated directly by plasma membrane budding and are in the range of 100–1000 nm in diameter, can initiate various biochemical pathways in MM cells, including MAPK and NF-kB signaling [15,16]. 

BMSCs are nonhematopoietic, multipotent progenitor cells able to differentiate into adipocytes, chondrocytes and osteocytes. BMSCs can be genetically and functionally altered during MM progression. Indeed, compared to their normal counterparts, MM-patient derived BMSCs exhibit much lower proliferative capacity and production of distinct growth and angiogenic factors [5,17,18,19]. Despite the enormous efforts to understand how the crosstalk between BMSCs and myeloma cells contributes to the evolution of this disease, our knowledge on the possible impact of BMSCs on the mechanisms regulating immune-mediated recognition and attack of MM cells remains unexplored. 

Natural killer (NK) cells are key effectors of the immune response against MM. Among NK cell activating receptors involved in recognition and killing of MM, a prominent role is played by the activating receptor DNAX accessory molecule-1 (DNAM-1/CD226), able to bind to two adhesion molecules of nectin family of proteins, nectin2 (Nect-2/CD112) and poliovirus receptor (PVR/CD155) [20,21,22], on MM cells. Although constitutively expressed on most normal tissues, Nectin-2 and PVR are found upregulated on tumor cells [23,24,25,26,27]. Moreover, both these ligands are capable of interacting with the inhibitory receptors TIGIT and tactile/CD96 also expressed by NK cells [28]. Indeed, these receptors play an important role in counterbalancing DNAM-1-mediated NK cell activation in the context of tumor microenvironment. 

Multiple myeloma cells have developed a number of strategies to evade immunosurveillance and detection by NK cells [29]. Thus, understanding the molecular mechanisms underlying the expression of DNAM-1 ligands on MM cells is crucial for the development of therapeutic approaches aimed at rendering these cancer cells more susceptible to recognition and killing by NK cells [30]. Our group has described different pathways involved in the transcriptional and post-translational regulation of DNAM-1 ligands in MM cells. IKZF1 and IKZF3 transcription factors repress PVR gene expression, while reactive oxygen species (ROS) and reactive nitrogen species (RNS) enhance PVR transcription via DNA damage response-E2F1 activation [31,32,33,34]. Moreover, sumoylation of PVR and ubiquitination of nectin2 are post-translational modifications responsible for protein intracellular retention and reduce MM cell susceptibility to NK cell cytotoxicity [35,36]. 

In this study, we demonstrate that PVR surface expression on MM cells is significantly enhanced by soluble factors produced by BMSCs via activation of NF-kB pathway. Of note, IL-8-bearing MVs released by BMSCs are essential for PVR upregulation and emerged as novel important regulators of this ligand. Our findings provide evidence of a role for BMSCs in NK cell anti-MM response and identify novel signaling events governing DNAM-1 ligand expression in cancer cells. 

## 2. Results

### 2.1. BMSC-Derived Soluble Factor(s) Increase PVR Surface Expression on MM Cells

We examined whether BMSCs could affect DNAM-1 ligand expression on MM cells thus impacting their recognition and susceptibility to NK cell-mediated lysis. To this aim, BMSCs were isolated from mononuclear cells obtained from BM aspirates of newly diagnosed MGUS (monoclonal gammopathy of undetermined significance) and active-MM patients. BMSCs were phenotypically characterized by flow cytometry assay by using a combination of monoclonal antibodies (Abs) (Figure S1A). According to the ISCT definition criteria [37,38], BMSCs lack the hematopoietic marker CD45, but express CD105, CD73, CD90, CD146, and CD106. Moreover, BMSCs capacity to differentiate in osteocytes and adipocytes was also evaluated (Figure S1B,C) and it was similar in the different disease stages.

**Figure 1:**
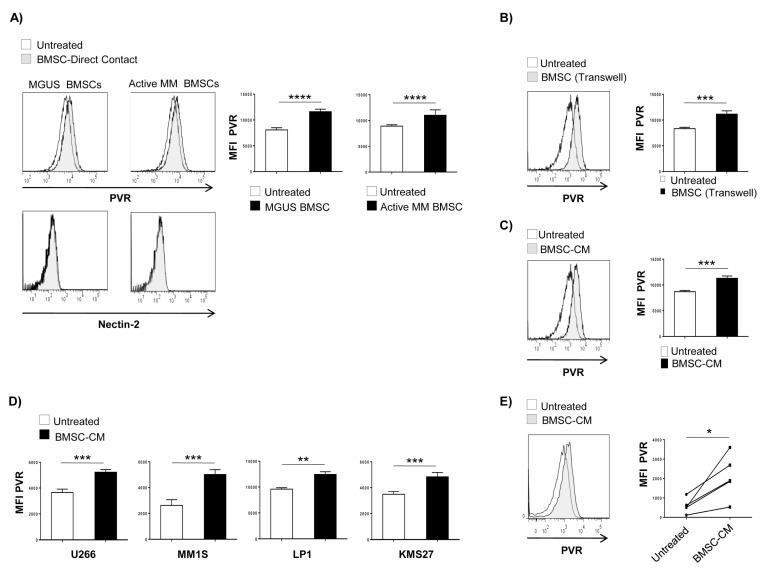
BMSC-derived soluble factor(s) increase PVR expression on human MM cells. PVR and Nectin-2 surface expression was analysed by flow cytometry on SKO-007(J3) cells co-cultured for 72 h in direct contact with BMSCs derived from MGUS or Active MM patients (**A**), using a transwell support (**B**) or BMSC-CM (**C**), and on the indicated MM cell lines (**D**) or on primary myeloma cells treated with BMSC-CM (**E**). For MM cell lines, histograms represent the MFI of specific mAb-MFI of isotype control. Data show mean ± SD calculated based on at least three independent experiments. For patient-derived PCs cells, data from 5 MM patients analysed are shown in right panel where each dot represents a single patient and indicates the MFI of specific mAb-MFI of isotype control. (* *p* < 0.05; ** *p* < 0.005; *** *p* < 0.002; paired Student *t*-test).

To analyze the possible effects of BMSCs on DNAM-1 ligand surface expression on MM cells, CFSE-stained SKO-007(J3) myeloma cells were seeded on a confluent monolayer of MGUS or active MM-BMSCs. After 72 h, PVR and nectin-2 expression on MM cells was analysed by immunofluorescence and flow cytometry. We observed that co-culture of SKO-007(J3) MM cells with BMSCs upregulates their basal membrane expression of PVR with no significant effects on Nectin-2 levels, which are undetectable on these cells (Figure 1A). Moreover, increased expression was observed for both MGUS and active MM-BMSCs, thus indicating that the capability of these cells to increase PVR expression on MM cells was independent on the disease stage. Based on these observations, we used only active MM-BMSCs in all other experiments. 

To discriminate between the contribution of direct cell-cell contacts and/or soluble factors in the BMSCs-induced PVR upregulation, we performed transwell experiments in which SKO-007(J3) MM cells were placed in the upper chamber and BMSCs or no cells (complete RPMI1640 medium alone) were placed in the lower chamber (Figure 1B). We observed augmented surface levels of PVR on SKO-007(J3) cells when co-cultured with BMSCs even in the presence of this insert that allows only diffusion of soluble factors. These findings demonstrate that BMSC-derived soluble factors enhance the expression of PVR activating ligand on MM cells. Accordingly, similar results were obtained by culturing SKO-007(J3) cells in conditioned medium (BMSC-CM) collected from 72 h culture of confluent BMSCs (Figure 1C). The possibility that these effects could be due to toxic and no specific effects of BMSC-CM on MM cells was excluded as these treatments did not affect cell viability over the time chosen for these experiments (as assessed by annexin V and propidium iodide staining) and the expression of MHC class I on SKO-007(J3) cells (Figure S2A,B). We then extended our analysis to additional MM cell lines and primary myeloma cells. We found that treatment with BMSC-CM was able to increase the basal expression of PVR on U266, KMS27, LP1, JJN3 and MM1S cell lines (Figure 1D) as well as on MM patient-derived PCs (Figure 1E and Table 1). All together, these results indicate that BMSCs are able to upregulate PVR expression on MM cells, and soluble factors play an important role in these mechanisms.

**Figure 2:**
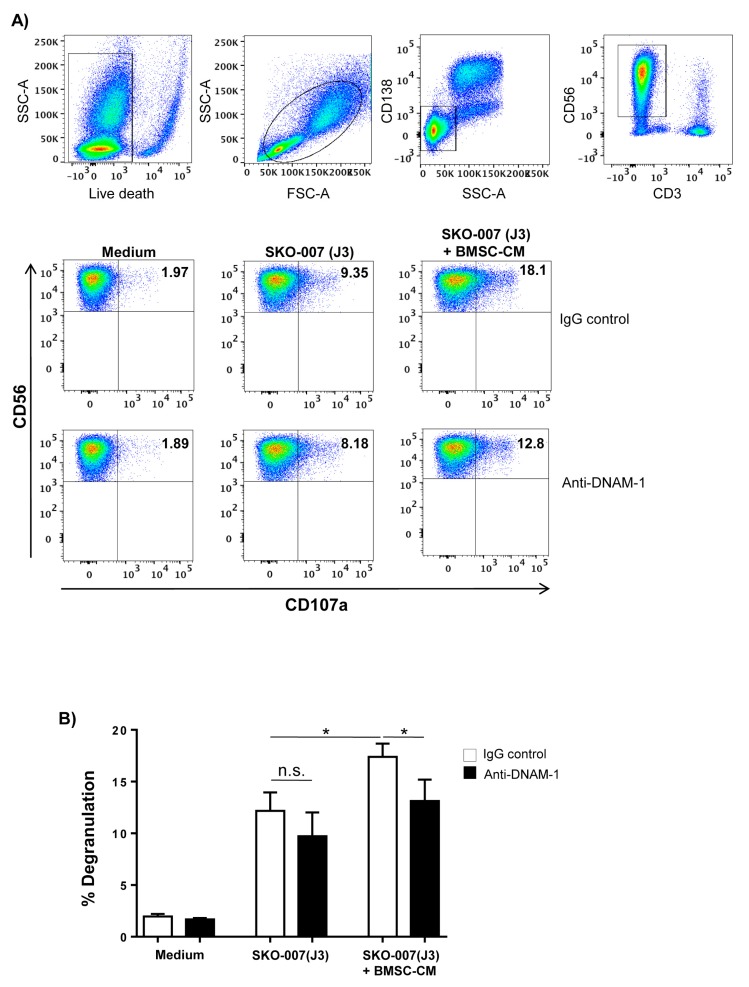
BMSCs enhance the ability of MM cells to stimulate NK cell degranulation by promoting DNAM-1 recognition. NK cells derived from PBMCs of healthy donors were incubated with SKO-007(J3) cells, untreated or cultured for 72 h with BMSC-CM and used as target cells in a degranulation assay. The assay was performed at the effector-target (E/T) ratio of 2.5:1. Cell surface expression of CD107a was analysed on CD3^−^/CD138^−^/CD56⁺ NK cells. The gating strategy used in flow cytometry analysis is shown in (**A**). To evaluate the role of DNAM-1, the assay was performed in parallel treating NK cells with blocking anti–DNAM-1 or anti-IgG mAbs used as control. Results are expressed as the percentage of CD107a^+^ cells. A representative experiment is shown. (**B**) Histogram represents the mean ± SD from three independent experiments (* *p* < 0.05, ANOVA).

To evaluate whether culture of MM cells with BMSC-CM could also potentiate NK cell degranulation, we then performed degranulation assays. As shown in Figure 2A,B, basal expression of CD107a on NK cells contacting SKO-007(J3) cells was enhanced following treatment with BMSC-CM. Increased degranulation was DNAM-1 dependent, because it was significantly reduced in the presence of a blocking anti-DNAM-1 mAb. 

**Figure 3:**
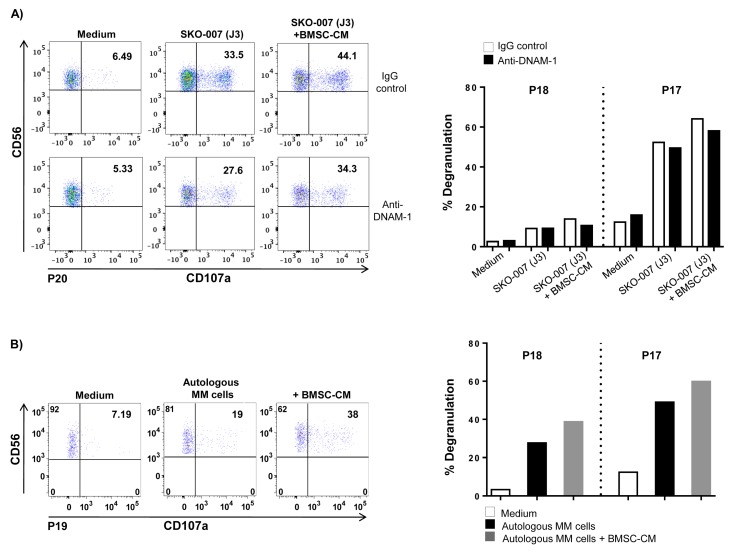
Patient-derived NK cell degranulation against BMSC-treated MM cells. CD138^−^ bone marrow cells were incubated with SKO-007(J3) (**A**) or autologous primary myeloma cells (**B**) untreated or treated with BMSC-CM for 72 h and used as target cells in a degranulation assay. The assay was performed at the effector: target (E:T) ratio of 2.5:1. After 3 h at 37 °C, cells were stained with anti-CD45, anti-CD138, anti-CD56, anti-CD3 and anti-CD107a mAbs. Cell surface expression of CD107a was analysed on CD56^+^CD3^−^CD138^−^ cells. In order to evaluate the role of DNAM-1, the assay was performed in parallel treating NK cells with blocking anti-DNAM-1 or isotype control antibodies. Results obtained from three patients for each condition (P17, P18 and P20 in A; P17, P18 and P19 in B) are shown.

Similarly, a higher extent of degranulation was also observed in patient-derived NK cells against SKO-007(J3) or autologous CD138+ plasma cells treated with BMSC-CM (Figure 3).

Overall, our results demonstrate that increased expression of PVR on MM cells cultured in the presence of BMSC-CM enhances NK cell degranulation by promoting their recognition by DNAM-1 activating receptor. 

### 2.2. Transcriptional Control of PVR Expression on MM Cells by BMSCs: Role of the Transcription Factor NF-kB

**Figure 4:**
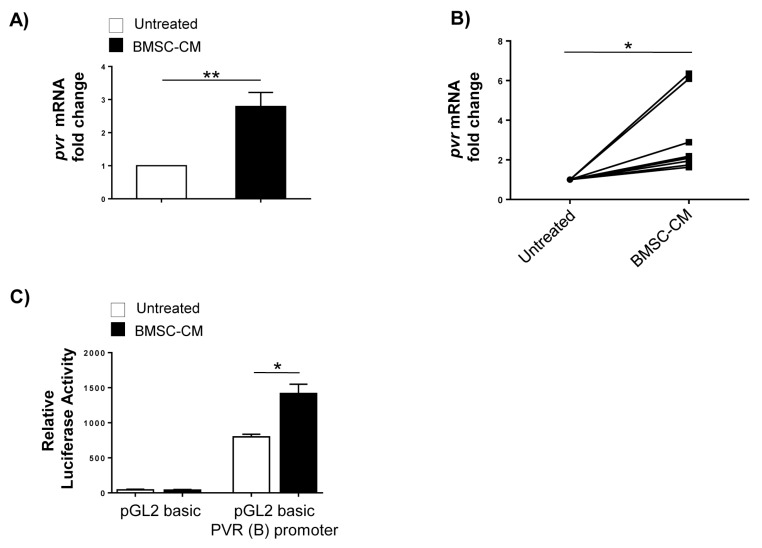
BMSC-CM increases PVR mRNA expression and promoter activity in MM cells. Real Time PCR analysis of total mRNA obtained from SKO-007(J3) cells (**A**) or patient-derived PCs (**B**) after 48 h stimulation with BMSC-CM or complete RPMI1640 medium. Data, expressed as fold change units, were normalized with GAPDH and referred to the untreated cells, considered as calibrator. For SKO-007(J3) cells, histograms represent the mean ± SD from three independent experiments. For primary myeloma cells, data from nine MM patients are shown where each dot represents a single patient. (* *p* < 0.05; ** *p* < 0.005; paired Student *t*-test). (**C**) SKO-007(J3) cells were transiently transfected with pGL2 basic- empty vector/PVR (B) promoter plasmid as described in materials and methods. After 48 h of treatment with BMSC-CM, SKO-007(J3) cells were harvested and protein extracts were prepared for the luciferase assay. Data, expressed as relative luciferase activity, were normalized to protein concentration and renilla activity and represent the mean ± SD from three independent experiments (* *p* < 0.05; paired Student *t*-test).

To establish whether BMSCs could regulate PVR expression at the transcriptional level, total RNA was isolated from SKO-007(J3) MM cells cultured in complete RPMI1640 medium or in the presence of BMSC-CM for 48 h and analysed by real-time quantitative RT-PCR. As shown in Figure 4A,B, we found a significant increase of *pvr* mRNA levels in SKO-007(J3) cells cultured in BMSC-CM, as well as in BMSC-CM-treated malignant PCs isolated from MM patients. We then transiently transfected PVR gene promoter in SKO-007(J3) cells to determine its transcriptional activity in response to BMSC-CM treatment. As shown in Figure 4C, BMSC-CM enhanced the activity of the reporter gene driven by a 343 bp fragment (B) of the *pvr* promoter. Collectively, these data indicate that BMSC-derived soluble factors increase PVR mRNA expression and promoter activity in MM cells.

**Figure 5:**
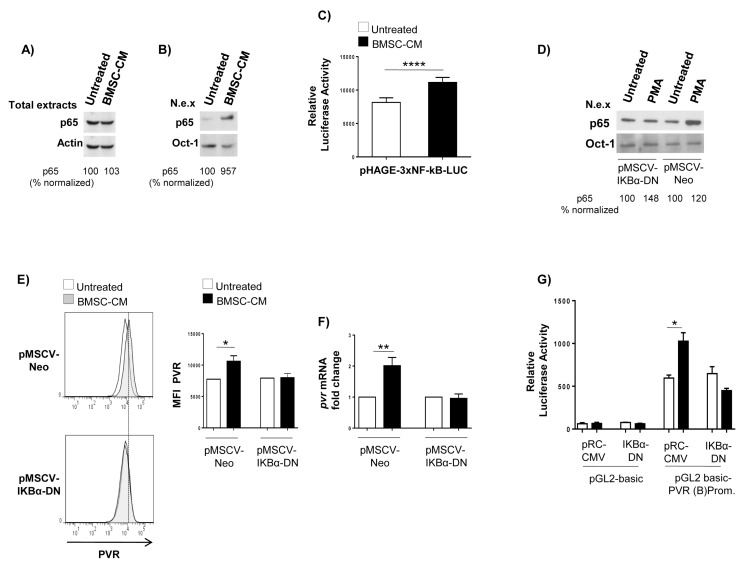
IKBα-DN blocks the induction of PVR expression by BMSC-CM. Western Blot analysis of total (**A**) or nuclear extracts (N.e.x.) (**B**) obtained from SKO-007(J3) cells untreated or stimulated for 24 h by BMSC-CM or (**D**) from SKO-007(J3) cells infected with pMSCV retroviral empty vector [pMSCV-Neo] or encoding a dominant negative form of IKBα (IKBα-DN) [pMSCV-IKBα-DN] untreated or treated for 30 min with PMA (10 ng/mL). The proteins transferred to nitrocellulose membranes were immunoblotted for p65 and actin or Oct-1. Numbers represent densitometric analysis of p65 normalized to actin or Oct-1 relative to the control untreated cells. (**C**) SKO-007(J3) cells were infected with lentivirus pHAGE-3xNF-kB-LUC-GFP obtained as described in Materials and Methods. After 72 h culture in BMSC-CM, SKO-007(J3) cells were harvested and analysed for luciferase activity. Results are expressed as relative luciferase activity and represent the mean ± SD from 4 independent experiments (**** *p* < 0.001; paired Student *t*-test). (**E**) Flow cytometric and (**F**) real time PCR analysis of PVR expression in SKO-007(J3) cells transduced with pMSCV-Neo or pMSCV-IKBα-DN, untreated or stimulated with BMSC-CM. For PVR surface expression, histograms represent the MFI of specific mAb-MFI of isotype control. The MFI of PVR was calculated based on at least 3 independent experiments ± SD (* *p* < 0.05; paired Student *t*-test). For PVR mRNA, data expressed as fold change units, were normalized with GAPDH and referred to the untreated cells, considered as calibrator and represent the mean of 3 experiments (** *p* < 0.05; * *p* < 0.05; paired Student *t*-test). (**G**) SKO-007(J3) cells were transiently co-transfected with pGL2 basic/PVR (B) promoter plasmid plus pRC-CMV empty vector or IKBα-DN and analysed for luciferase activity as described above. Results are expressed as relative luciferase activity and represent the mean ± SD from four independent experiments (* *p* < 0.05; paired Student *t*-test).

To identify possible molecular mediators involved in the upregulation of PVR by BMSCs, we focused our attention on NF-kB family transcription factors. These proteins are constitutively active in MM cells but a number of studies demonstrated increased NF-kB activity in MM cells in response to soluble factors produced by BMSCs [9,39,40,41]. Despite no evidence is available about a role of this transcription factor in the regulation of PVR gene expression in cancer cells, a search for sequence homology revealed the presence of putative NF-kB binding sites (-254-GGGGAGGGCCAG-243; and -162-GTGGGTATTCCC-150) in the region of PVR(B) promoter spanning −343 bp from the transcription start site. We first confirmed the capability of BMSC-CM to induce NF-kB activation in MM cells in our experimental setting. As nuclear translocation is an important step leading to NF-kB activation, total and nuclear extracts were prepared from SKO-007(J3) cells cultured for 24 h in BMSC-CM or complete RPMI1640 medium and were analysed by western blotting assay. We observed that treatment with BMSC-CM does not affect total amount of NF-kB subunit p65 (Figure 5A) but can induce nuclear translocation of this protein (Figure 5B). Then, we evaluated the possible effects of BMSC-CM on the activity of this transcription factor. To this purpose, we infected SKO-007(J3) cells with a lentivirus expressing a NF-kB-responsive luciferase construct (pHAGE-3xNF-kB-LUC-GFP). As shown in Figure 5C, we found that culture of MM cells with BMSC-CM for 72 h significantly enhances luciferase expression thus indicating an increased NF-kB transcriptional activity. Overall, these findings indicate that BMSC-CM can induce NF-kB pathway activation in MM cells.

To examine the role of NF-kB in PVR regulation by BMSCs, we transduced a dominant negative form of IKBα (pMSCV-Neo-IKBα-DN), the cytoplasmic repressor of NF-kB, by retroviral infection in SKO-007(J3) cells. After selection in neomycin, the IKBα-DN overexpression was demonstrated in western blotting assays. As shown in Figure 5D, treatment with a known NF-kB inducer, phorbol myristate acetate (PMA), was able to induce p65 nuclear translocation in pMSCV-Neo infected SKO-007(J3) cells but not in pMSCV-Neo-IKBα-DN overexpressing cells. Flow cytometric and Real-Time RT-PCR analysis on these cells revealed that IKBα-DN overexpression blocked BMSC-CM-induced increase of PVR surface expression and mRNA levels (Figure 5E,F). Accordingly, the activity of PVR (B) promoter fragment was also reduced when BMSC-CM-treated cells were co-transfected with IKBα-DN expression vector, whereas no difference was observed when the empty control vector was used (Figure 5G). Taken together, these findings demonstrate that NF-kB can act as an activator of PVR expression on MM cells in response to soluble factors secreted by BMSCs. 

### 2.3. Increased PVR Expression on MM Cells by BMSCs Involves IL-8

Analysis of microarray public data of MM patients (datasets GSE2658, GSE6477 available at http://www.ncbi.nlm.nih.gov/geo/) indicates that a significant positive correlation exists between PVR and chemokine receptor expression, CXCR1 (R  =  0.335 in Hanamura MM Dataset of R2 platform for genomic analysis) and CXCR2 (R  =  0.239 in Chng MM Dataset of R2 platform for genomic analysis (Figure S3A,B). 

Based on these observations, to gain further insight into the nature of the BMSC-secreted factors capable of inducing PVR expression on MM cells, we focused our attention on CXCR1/2 ligand CXCL8/IL-8, a chemokine produced by BMSCs and able to trigger NF-kB activation in MM cells [39]. We first confirmed the capability of BMSCs to secrete IL-8 in our experimental setting. We collected cell-free supernatants from BMSCs after 72 h culture and tested them for the presence of this cytokine by ELISA. We observed that IL-8 is expressed by BMSCs with no significant difference between MGUS and Active MM BMSCs (Figure S3C and Table 2). Moreover, by FACS analysis we revealed that IL-8 receptors CXCR1 and CXCR2 are expressed on SKO-007(J3) cells and treatment with BMSC-CM does not change the levels of these proteins (Figure S3D).

To evaluate the possible contribution of IL-8 in PVR upregulation, we used two different approaches to block CXCR1 and CXCR2 activity. SKO-007(J3) cells were treated with an anti-CXCR1 blocking mAb or a selective CXCR2 antagonist, SB225002, before stimulation with BMSC-CM. 

**Figure 6:**
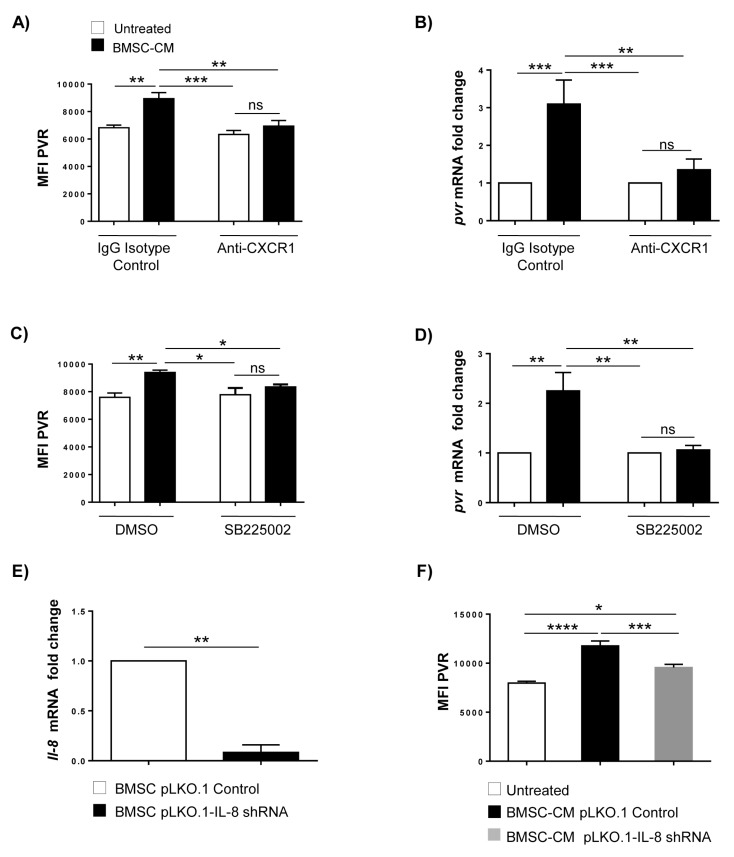
Blockade of CXCR1/2 receptors prevents PVR upregulation by BMSC-CM. PVR surface expression was analysed by flow cytometry. SKO-007(J3) cells were pre-treated with anti-CXCR1 or IgG control (**A**) or with SB225002 or vehicle control (DMSO) (**C**), and stimulated by BMSC-CM. Real Time PCR analysis of total PVR mRNA obtained from SKO-007(J3) cells after 48h stimulation with BMSC-CM and pretreatment with anti-CXCR1 blocking mAb (**B**) or SB225002 (**D**) as described above. (**E**) Real Time PCR analysis of total IL-8 mRNA obtained from BMSCs infected with lentiviral vector expressing IL-8 shRNA (pLKO.1-IL-8 shRNA) or scrambled control pLKO.1 control. Data, expressed as fold change units, were normalized with GAPDH and referred to the untreated cells, considered as calibrator. Data were calculated based on at least 3 independent experiments ± SD (* *p* < 0.05; *** *p* < 0.002; **** *p* < 0.001; ANOVA). (**F**) Cytofluorimetric analysis of PVR surface expression of SKO-007(J3) cells treated for 72 h with conditioned medium derived from BMSCs transduced with pLKO.1-IL-8 shRNA or scrambled control pLKO.1 control. Histograms represent the MFI of specific mAb-MFI of isotype control. Data were calculated based on at least three independent experiments ± SD (* *p* < 0.05; *** *p* < 0.002; **** *p* < 0.001; ANOVA).

We observed that both treatments significantly block PVR upregulation at protein (Figure 6A,C) and mRNA level (Figure 6B,D). Consistently, a significant reduction of PVR upregulation was observed in SKO-007(J3) cells treated with conditioned medium obtained from IL-8 silenced-BMSCs (Figure 6E,F). Altogether, these data identify the BMSC-derived IL-8 as a novel regulator of PVR expression in MM cells. 

### 2.4. Requirement of IL-8-Bearing Microvesicles for BMSC-Induced PVR Upregulation

Recent studies demonstrated that microvesicles (MVs) are crucial mediators of intercellular communication between MM and BMSCs. Indeed, BMSC-derived MVs can activate different signaling pathways, including NF-kB, in MM cells [15,16]. Since MVs are also known to encapsulate or bind on their surface different cytokines [42], we examined the possible role of MVs in the regulation of PVR expression by BMSCs. To this aim, we isolated extracellular vesicles by BMSC-CM via ultracentrifugation and validated their identity by transmission electron microscopy and DLS experiments that demonstrated membrane vesicles of size 200–1000 nm resembling MVs (Figure S4A,B). Flow cytometry analysis showed that BMSC-derived MVs are negative for the hematopoietic marker CD45, but they bear the surface molecules typically expressed by BMSCs, such as CD90, CD105 and CD73 (Figure S4C).

**Figure 7:**
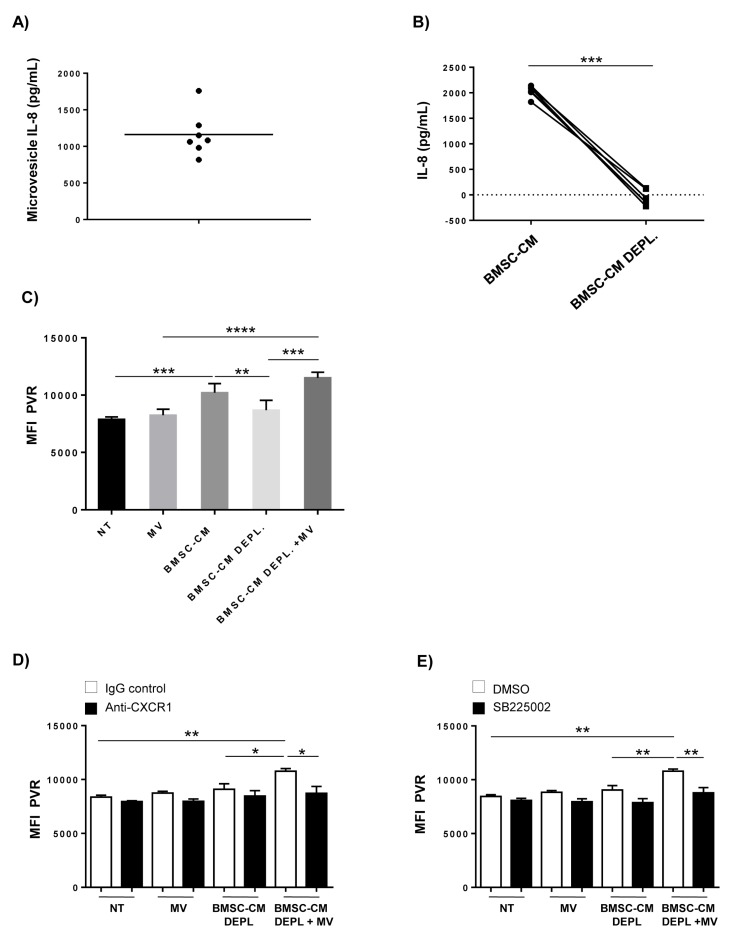
Role of BMSC-derived microvesicles bearing IL-8 in the induction of PVR expression on MM cells. (**A**) Whole BMSC-derived MVs (**B**) or complete and BMSC-CM lacking MVs (BMSC-CM DEPL.) were analysed for IL-8 by ELISA assay. Data were calculated based on at least 3 independent experiments ± SD (* *p* < 0.05; paired Student *t*-test). (**C**) PVR surface expression was analysed by flow cytometry on SKO-007(J3) cells cultured for 72 h in complete or microvesicle-depleted BMSC-CM (BMSC-CM DEPL.) alone or in combination with stromal MVs (10 µg), in the absence or (**D**) in the presence of a blocking anti-CXCR1 or (**E**) a CRCX2 antagonist, SB225002 as described above. Histograms represent the MFI of specific mAb-MFI of isotype control. Data were calculated based on at least three independent experiments ± SD (* *p* < 0.05; ** *p* < 0.005; *** *p* < 0.002; ANOVA).

We then performed ELISA for IL-8 by using whole MVs obtained from BMSCs. We revealed the presence of IL-8 on MVs surface (Figure 7A), thus suggesting the involvement of vesicles bearing this cytokine in the induction of PVR expression. Accordingly, IL-8 was almost eliminated from BMSC-CM following depletion of MVs by ultracentrifugation and filtration (BMSC-CM DEPL.) (Figure 7B). Moreover, when we evaluated the presence of IL-8 in two different fractions of BMSC-CM, containing molecules with molecular weight higher or lower than 100 KDa, we found that the fraction >100 KDa contained the majority of IL-8 and was also responsible for PVR upregulation (Figure S5A,B). These findings indicate that despite its very low molecular weight (8 KDa), IL-8 is an important component of the fraction >100 KDa of BMSC-CM, that is consistent with the association of the chemokine with MVs.

Based on these observations, we evaluated the possible effects of BMSC-derived MVs in PVR upregulation. We observed that the combined treatment of SKO-007(J3) cells with BMSC-CM DEPL and MVs enhance PVR levels as well as complete BMSC-CM (Figure 7C) and an anti-CXCR1 blocking mAb or a selective CXCR2 antagonist, SB225002, abolished PVR induction (Figure 7D,E), thus indicating a role for IL-8 in mediating these effects. 

However, when used alone neither BMSC-derived MVs or BMSC-CM DEPL were able to increase PVR surface expression, thus indicating a cooperative activity of IL-8/MVs with other (s) soluble factor(s) produced by BMSCs. In particular, we cannot exclude that other vesicles, such as exosomes, may be involved in these mechanisms and further investigations are needed to address this possibility.

Taken together, these results demonstrate that IL-8 is associated with bone marrow stromal MVs which are also required for PVR upregulation via CXCR1/CXCR2 signaling activation; moreover, they indicate that MVs are necessary but not sufficient per sè to enhance PVR expression.

## 3. Discussion

Several lines of evidence described the capability of NK cells to recognize and attack malignant PCs indicating a role for these lymphocytes in the control of MM development. In particular, the engagement of DNAM-1 activating receptor plays an important role in the recognition and killing of MM cells [20,21,22,23]. Thus, understanding the mechanisms responsible for the regulation of DNAM-1 ligands on MM cells will be useful to address strategies to enhance their susceptibility to NK cell-mediated attack. In this study, we investigated the possible role of MM BM microenvironment in these mechanisms focusing on the interaction between MM cells and BMSCs. 

It is well known that BMSCs can be genetically and functionally altered during MM progression and promote tumor cell survival and proliferation by direct contact with MM cells as well as by secretion of numerous soluble factors [6,43]. In this context, here we added novel findings of a significant upregulation of PVR mRNA and cell surface expression on MM cell lines as well as on patient-derived PCs by BMSCs, thus enhancing their recognition and NK cell degranulation via engagement of DNAM-1 activating receptor. Of note, we did not find any difference between BMSCs isolated from MGUS or active MM patients, suggesting that the capability of BMSCs to induce the expression of PVR on MM cells is not affected by tumor context and it is not a property of BMSCs at a specific disease stage.

Mechanistically, BMSC-derived soluble factor(s) are key mediators of increased surface PVR levels on MM cells; indeed, we found the upregulation of this ligand by performing direct contact co-culture as well as through transwell support or culturing MM cells in BMSC-CM. Moreover, the observation that BMSC-CM is sufficient per sè to fully induce PVR expression indicates that soluble factor(s) mediating these effects are constitutively produced by BMSCs. 

Of note, we identified the chemokine IL-8 as a critical mediator of PVR upregulation on MM cells by BMSCs. We observed that PVR expression was selectively reduced both at level of mRNA and protein upon blockade of CXCR1/2 receptors on myeloma cells as well as by depletion of IL-8 via shRNA in BMSCs. 

IL-8 acts as an important activator of BMSC-induced NF-κB activity in MM cells and the consequent resistance to bortezomib [39]. Consistently, our findings demonstrate that the transcription factor NF-kB is critically involved in PVR regulation by BMSCs. We showed that IKBα-DN overexpression inhibits PVR upregulation by BMSC-CM. Interestingly, despite constitutive NF-kB activation in MM cells, basal expression of the ligand was not affected. These findings indicate that PVR upregulation by BMSCs may require the cooperative action of NF-kB proteins and other transcription factors which remain to be defined. To date, the role of NF-kB in these regulatory mechanisms in tumor cells has never been studied. A search for sequence homology revealed the presence of putative NF-kB binding sites (-254-GGGGAGGGCCAG-243; and -162-GTGGGTATTCCC-150) in the region of PVR(B) promoter spanning −343 bp from the transcription start site with increased activity in BMSC-CM treated cells. The possible contribution of these regulatory elements in BMSC-mediated PVR upregulation will be examined in future studies. 

A very interesting and novel aspect of the regulatory mechanisms emerging in this study regards the role of MVs. Many cytokines can be released by cells in encapsulated or associated form with extracellular vesicles and are able to elicit biological effects upon contact with sensitive cells. However, a given cytokine can be released predominantly in a soluble form or vesicle-associated in a cell-dependent way [42]. We found that the majority of IL-8 is associated with MVs derived from BMSCs. It may be interesting to understand if exosomes may also associate IL-8 and exert the same effects as MVs.

Even though MVs endowed with IL-8 are not sufficient alone to upregulate PVR expression, they are necessary for this mechanism suggesting a cooperative activity of IL-8/MVs with other (s) soluble factor(s) produced by BMSCs. Further studies are needed for their identification. 

IL-8 may be required for triggering and/or sustaining the signaling of one or more distinct soluble factors involved in PVR regulation. To this regard, there is evidence of a crosstalk between receptors of IL-8 and growth factors in endothelial and cancer cells. IL-8 activation of CXCR1/2 can transactivate growth factor receptors, such as EGFR or VEGFR, via direct interaction or receptor phosphorylation by prolonging and reinforcing their signaling and cellular responses [44,45,46,47]. Alternatively, CXCR1/2-IL-8 interaction may be promoted by other(s) soluble factor(s) released by BMSCs. Consistently, other ligands and chemokine receptors can affect the functions of CXCR1 and CXCR2. For instance, the atypical receptor CCRL2 is required for CXCR2-dependent neutrophil recruitment; in this context, CCRL2 does not bind IL-8 but forms heterodimers with CXCR2 and regulates IL-8 induced-CXCR2 signaling [48]. 

Although PVR upregulation results in the promotion of NK effector activity against MM cells, because BMSC-derived IL-8 can also increase the expression of different adhesion molecules, such as VCAM-1 and ICAM-1 [39], it cannot be rule out that PVR upregulation on MM cells may support their survival and proliferation by enhancing the adhesion to BMSCs. Moreover, another scenario of IL-8-induced PVR regulation may be linked to the ability of this ligand to bind to both activating and inhibitory receptors thus leading to increased or reduced activity of immune effector cells. Indeed, an important mechanism of immune evasion involves decreased activating DNAM-1 and increased inhibitory TIGIT/CD96 expression on tumor infiltrating immune cells. To this regard, DNAM-1 expression is downregulated on NK cells from MM patients with active disease compared to patients in remission [49]. Taking in consideration these findings, we could hypothesize that PVR overexpression renders MM cells more susceptible to NK cell mediated attack at early stage of diseases, while leading to a defective NK cell activity during disease progression when receptor repertoire of these cytotoxic lymphocytes results altered and the interaction PVR/TIGIT or PVR/CD96 may play a dominant role in the BM MM microenvironment. Consistently, PVR overexpression was correlated with tumor progression and unfavorable prognosis in different cancer cell types [50,51,52].

## 4. Materials and Methods

### 4.1. Cell Lines and Clinical Samples

Human MM cell line SKO-007(J3) was kindly provided by Prof. P. Trivedi (Sapienza, University of Rome, Rome, Italy). The human MM cell lines JJN-3, KMS-27, MM1S, and LP1 were kindly provided by Prof. Nicola Giuliani (University of Parma, Parma, Italy). MM cell lines were maintained at 37 °C and 5% CO_2_ in RPMI 1640 (ThermoFisher Scientfic, Waltham, MA, USA) supplemented with 10% FCS. The human 293T embryonic kidney cells were purchased from ATTC (Manassas, VA, USA) and were maintained in Dulbecco’s modified Eagle’s supplemented with 10% FCS. All cell lines were mycoplasma-free (EZ-PCR Mycoplasma Test Kit, Biological Industries, Kibbutz Beit-Haemek, Israel). 

Bone marrow (BM) samples from MM patients were managed at the Division of Hematology, Department of Translational Medicine and Precision, Sapienza University of Rome. Informed consent in accordance with the Declaration of Helsinki was obtained from all patients, and approval was obtained from the Ethics Committee of the Sapienza University of Rome (RIF.CE: 5191). BM aspirates were processed as described in [23]. MM cells were selected using anti-CD138 magnetic beads (Miltenyi Biotec, Auburn, CA, USA). More than 95% of the purified cells expressed CD138 and CD38. CD138 negative fraction was harvested to obtain bone marrow stromal cells (BMSCs). Cells were plated (1 × 10^6^ per cm^2^) in MEMα medium supplemented with 15% FBS, 2 mM L-glutamine, 100 U/mL penicillin and 100 U/mL streptomycin at 37 °C and 5% CO₂. After 24 h, non-adherent cells were removed to select only adherent cells. In keeping with the International Society for Cellular Therapy (ISCT) recommendations [37,38], a combination of antibodies, including anti-CD45/APC-H7, anti-CD90/PeCy5, anti-CD105/APC, anti-CD146/BV395, anti-CD73/V450 PEA, anti-CD106/PE, was used to phenotypically characterize BMSCs by a multiparameter flow cytometry analysis. The fluorescence was analysed using a FACS LRSFORTESSA flow cytometer (BD Biosciences, San Jose, CA, USA) and FlowJoV10 (Treestar Inc, Ashland, OR, USA) software. BMSCs were used only until fifth passage.

### 4.2. Adipogenic and Osteogenic Differentiation

For adipogenic differentiation, 1 × 10^5^ BMSCs were seeded on 6-well plates and cultured for 14 days with adipogenic induction medium containing 1 µmol/L dexamethasone, 0.5 mmol/L isobutylmethylxanthine and 100 µmol/L indomethacin. Cells cultured in MEMα medium supplemented with 15% FBS served as a negative control. Adipogenic differentiation was assessed by Oil-Red-O staining (Sigma Aldrich, St. Louis, MO, USA) according to the manufacturer instructions and by real-time PCR analysis of PPAR-γ1 and PPAR-γ2 gene expression. 

For osteogenic differentiation, BMSCs were seeded at 5 × 10^3^/cm^2^ on 12-well plates and cultured for 21 days in osteogenic induction medium containing 0.1 µmol/L dexamethasone, 10 mmol/L β-glycerol phosphate, and 200 µmol/L ascorbate-2-phosphate. Cells cultured in MEMα medium supplemented with 15% FBS were used as a negative control. Osteogenic differentiation was detected by von Kossa staining (Sigma Aldrich) according to the manufacturer instructions and by real-time PCR analysis of osteopontin and Runx2 gene expression. 

### 4.3. Reagents and Antibodies

The selective CXCR2 antagonist SB225002 and 7-AAD were purchased from Sigma-Aldrich. Monoclonal antibodies (mAbs) anti-CD138/FITC, anti-CD38/APC, anti-CD107a/APC, anti-CD3/FITC, anti-CD56/PE, anti-CD45/APCH7, anti-CD90/PeCy5, anti-CD105/APC, anti-CD146/BV395, anti-CD106/PE and anti-CD73/V450 were purchased from BD Biosciences. mAb anti-CXCR2/PE was purchased from Miltenyi Biotec. mAb anti-DNAM-1 (DX11) was purchased from Serotec-Biorad (Hercules, CA, USA). Conjugate anti-PVR/PeCy7 and anti-CXCR1 (MAB330-SP) were purchased from R&D Systems (Minneapolis, MN, Canada). Anti-PVR (SKII.4) was kindly provided by Prof. M. Colonna (Washington University, St Louis, MO, USA). Anti-MHC class I (W6/32) was purchased from ATCC. Allophycocyanin (APC)-conjugated with goat anti mouse antibody was purchased from Jackson Immuno-Research Laboratories (Cambridgeshire, United Kingdom).

### 4.4. Flow Cytometry and Degranulation Assay

MM cell lines were cultured in 24-well tissue culture plates for 72 h at a concentration of 1.5 × 10^5^ cells/mL in direct contact with BMSCs or in the presence of a transwell system or in BMSC-conditioned medium (BMSC-CM). Cells were stained with anti-PVR unconjugated mAb followed by secondary goat anti mouse allophycocyanin antibody. In direct-contact experiments, SKO-007(J3) cells were stained with CFSE before co-culture to discriminate MM cells from BMSCs by FACS analysis. In some experiments, SKO-007(J3) cells were pretreated for 1 h at 4 °C with anti-CXCR1 or IgG control (1 µg/10^6^ cells) or were pre-treated with for 1 h at 37 °C with a CRCX2 antagonist, SB225002 (12.5 nM) or vehicle control (DMSO). In all experiments, cells were stained with propidium iodide (PI, 1 µg/mL) in order to assess cell viability (always higher than 90% after the different treatments). Nonspecific fluorescence was assessed by using an isotype-matched irrelevant mAb (R&D Systems) followed by the same secondary antibody. Patient-derived plasma cells (2 × 10^6^ cells/mL) were cultured for 48h in BMSC-CM or complete RPMI1640 medium supplemented with IL-3 (20 ng/mL) and IL-6 (2 ng/mL). The membrane expression of PVR was analysed by immunofluorescence staining with anti-PVR/PeCy7 or matched isotype control. All samples were also stained with Fixable Viability Stain 450 (FVS450) (BD Biosciences) to discriminate cell viability. 

NK cell-mediated degranulation was determined by cell-surface expression of the lysosomal marker CD107a as previously described [53,54]. As source of effector cells, we used primary cultured NK cells obtained as previously described [55]. In experiments with patient-derived NK cells, CD138^−^ BM cells were used as source of effector cells.

SKO-007(J3) cells or patient-derived plasma cells cultured in BMSC-CM for 72 h were incubated with NK cells at 2.5:1 E:T ratio in complete medium at 37 °C and 5% CO_2_ for 2.5 h. Thereafter, cells were washed with PBS and incubated for 30 min at 4 °C with anti-CD107a/APC, anti-CD3/FITC, anti-CD56/PE and anti-CD138 PercP to gate the CD3^−^CD138^−^CD56^+^ NK cell population. All samples were also stained with APC-H7 (BD Biosciences) to discriminate cell viability. In some experiments, cells were pre-treated for 20 min at room temperature with anti-DNAM-1 neutralizing mAb Fluorescence was analysed using FACS Canto II flow cytometer (BD Biosciences). Flow cytometric analysis was performed using FlowJo Flow Cytometric Analysis Software.

NK cell cytotoxicity was evaluated by flow-cytometry analysis. SKO-007(J3) target cells were labeled with CFSE (1 µmol/L) and were incubated with NK cells at different E:T ratios in complete medium at 37 °C and 5% CO_2_. CFSE-labeled target cells alone were used as negative control in each test. After coculture for 4 h, the cell mixture was stained with 7-AAD (5 µg/mL) for 20 min, washed with PBS and fixed by PFA 1%. Fluorescence was analysed using FACS Canto II flow cytometer (BD Biosciences). NK cell cytotoxicity was calculated as cells positive for both CFSE and 7-AAD, after substracting the spontaneous lysis in negative control.

### 4.5. Western-Blot Analysis

To obtain whole cell extract, SKO-007(J3) cells were lysed for 30 min on ice in lysis buffer [1% Nonidet P-40 (*v*/*v*), 10% glycerol, 0.1% SDS, 0.5% sodium deoxycholate, 1 mM phenylmethyl-sulfonyl fluoride, 10 mM NaF, 1 mM Na3VO4, complete protease inhibitor mixture (Sigma Aldrich) in PBS]. Nuclear extracts were prepared from SKO-007(J3) cells as previously described [56,57]. Protein concentration was determined by the BCA method (ThermoFisher Scientfic, Waltham, MA, USA). Thirty to 50 μg was resolved by SDSPAGE and transferred to nitrocellulose membranes (Whatman GmbH, Dassel, Germany). After blocking in milk, membranes were probed with specific Abs. Antibodies against p65 and Oct-1 were purchased from Santa Cruz Biotechnology (Dallas, TX, USA)). Antibody against *β*-actin was purchased from Sigma Aldrich. An HRP-conjugated secondary Ab and an ECL detection system (Amersham, GE Healthcare, London, United Kingdom) were used to reveal immunoreactivity.

### 4.6. Plasmids

The fragment (B) −343 bp of the human PVR promoter cloned in pGL2-basic luciferase vector (Promega, Madison, WI, USA) [pGL2-basic-PVR(B) promoter plasmid] was kindly provided by Dr. Bernhardt G. (Hannover Medical School, Hannover, Germany) [58]. The expression vector coding for the dominant negative mutant of the repressor IKBα (S32A and S36A) in pRC-CMV was provided by A. Israel (Institut Pasteur Paris, Paris, France). The retroviral vector coding for the dominant-negative mutant of the repressor IKBα (S32A and S36A) in pMSCV-Neo (Murine Stem Cell Virus-Neomycin) was provided by J. Hischott (McGill University, Montreal, QC, Canada). The lentiviral vector pHAGE-3xNF-kB-LUC-GFP expressing the green fluorescence gene insert and containing the luciferase gene driven by NF-kB-responsive consensus sequences and the plasmids pVSG-5, psPAX2, pGAg-Pol-Env, and pTK-Green Renilla were all purchased from Addgene (Watertown, MA, USA)). For knocking down CXCL-8/IL-8, we used a pLKO.1-shCXCL-8 (TRCN0000369178) lentiviral vector with puromycin resistance and the control vector pLKO.1 non-targeting shRNA (MISSION™ Sigma-Aldrich). 

### 4.7. DNA Transfections, Virus Production and In Vitro Transduction

For virus production, HEK293 was transfected with 5 μg of viral DNA together with pVSV-5 and psPAX2 or pGAg-Pol-Env packaging vectors, using Lipofectamine 2000 (Life Technologies) as previously described [59]. SKO-007(J3) cells were infected as previously described [31]. 1 × 10^5^ BMSCs seeded on 6-well plate were infected with 2 mL viral supernatant in complete medium containing 8 μg/mL Polybrene (Sigma-Aldrich). Cells were allowed to expand for 24 h and were then selected for neomycin (1 mg/mL) or for puromycin (2 µg/mL) resistance for pMSCV-Neo retroviral vector or for pLKO.1 lentiviral vector, respectively. For GFP-expressing viruses (pHAGE-3xNF-kB-LUC-GFP), the infection efficiency was measured by FACS analysis of GFP expression at day 3 after infection. 

SKO-007(J3) cells were transfected with the plasmids using Amaxa nucleofection procedure (Lonza Bioscience, Basel, Switzerland) as already described [53] and treated with BMSC-conditioned medium. After 48 h, cells were collected, and protein extracts were prepared for the luciferase assay. A TK-renilla expression vector was co-transfected each time to normalize DNA uptake. Luciferase and renilla activity were read using Dual-Luciferase Reporter Assay and the Glomax Multi Detection System (Promega) following the manufacturer’s instructions.

### 4.8. RNA Isolation and Quantitative Real-Time Polymerase Chain Reaction (qRT-PCR)

Total RNA was extracted using the total RNA mini Kit following instructions provided by the manufacturer (Geneaid Biotech, New Taipei City, Taiwan) and 2 µg were used for cDNA first-strand synthesis in a 25 µL reaction volume according to the manufacturer’s protocol for M-MLV reverse transcriptase (Promega). 

PVR, IL-8 and GAPDH mRNA expression were analysed by real-time PCR using the following specific TaqMan Gene Expression Assays (Applied Biosystems): PVR (Hs00197846_m1), CXCL8/IL-8 (Hs00174103_m1), and GAPDH (Hs99999905_m1). 

cDNAs obtained from RNA derived by differentiated BMSCs were amplified in triplicate with primers for human osteopontin, Runx2, PPAR-γ1, PPAR-γ2 and for GAPDH by using the Power-SYBR green mix with ROX (Applied Biosystems). Primer sequences were as follows: human osteopontin forward: TTGCAGCCTTCTCAGCCAA; osteopontin reverse: GGAGGCAAAAGCAA ATCACTG; human Runx2 forward: ATGTGTGTTTGTTTCAGCAGCA; Runx2 reverse: TCCCTAA AGTCACTCGGTATGTGTA; human PPAR-γ1 forward: CTATGGAGTTCATGCTTGTG; PPAR-γ1 reverse: GTAC TGAGTACTGACA TTTATTT; human PPAR-γ2 forward: CGAGGACACCGG AGAGGG; PPAR-γ2 reverse: TGTGGTTTAGTGTTGGCTTCTT; human GAPDH forward: TCGAC AGTCAGCCGCATCT; GAPDH reverse: CCGTTGACTCCGACCTTCA. The level of expression was measured using Ct (threshold cycle). Relative expression of each gene versus the housekeeping gene was calculated according to the 2^−ΔΔCt^ method. The analysis was performed using the SDS version 2.4 software (Applied Biosystems).

### 4.9. Annexin V

Apoptotic cell death was evaluated using APC Annexin-V Apoptosis Detection Kit with PI (Thermo Fisher Scientific, Waltham, MA, USA). Briefly, 15 × 10^4^ SKO-007(J3) were cultured in 24-well plates, untreated or treated with BMSC-CM for 72 h. Cells were then stained using Annexin-V/APC and PI according to the manufacturer’s instruction. Cell populations were acquired using FACS Canto II flow cytometer (BD Biosciences). Flow cytometric analysis was performed using Flow Jo Flow Cytometric Analysis Software.

### 4.10. Enzyme-Linked Immunosorbent Assay (ELISA)

BMSC-conditioned medium and MVs derived from BMSCs were analysed for CXCL8/IL-8 by ELISA following instructions provided by the manufacturer (R&D Systems). BMSC-CM was collected from 72 h culture of 2 × 10^4^ BMSCs in 1 mL of serum-free medium in a 24 well plate. Analysis of MVs was performed in the absence of lysis buffer and PBS was used as a control. Absorbance was measured at 450/540 nm with Victor2 Microplate Reader (Perkin Elmer–GMI Waltham, MA, USA), and IL-8 concentration was calculated in correlation to a standard curve of control samples.

### 4.11. Preparation of BMSC-CM and Microvesicles

Medium collected from 72 h-culture of confluent BMSCs (2 × 10^4^ cells) (BMSC-CM) was used to stimulate MM cell lines for 72 h. BMSC-CM and complete RPMI1640 medium were separated in different fractions by Amicon Ultra-15 centrifugal filter devices (Merck Millipore, St.Louis, MO, USA) according to the manufacturer’s instructions.

For microvesicle isolation, MVs-free medium was obtained by centrifugation of FBS at 100,000× *g* for 3 h in a Beckman ultracentrifuge (Beckman Coulter, Brea, CA, USA). MEM-α medium was supplemented with 15% of FBS-MVs free and antibiotics. BMSCs cells were cultured at 1 × 10^6^ cells/mL in MV-free medium for 72 h. Conditioned media was first centrifuged at 800× *g* for 5 min to remove cells and then centrifuged at 4500× *g* for 5 min to discard large debris. After centrifugation at 20,000× *g* (Beckman Coulter) for 60 min at 4 °C, both supernatant and pelleted MVs were recovered. The supernatant was 0.22 µm filtered and used as conditioned medium lacking MVs (BMSC-CM Depleted). The MVs were washed and re-suspended in PBS and MV total protein concentration was measured at 280 nm using a NanoDrop spectrophotometer (Thermo Fisher Scientific).

Transmission electron microscopy (TEM) of BMSCs-isolated MVs was performed as following described. Briefly, MVs were fixed in 2% PFA and adsorbed on formvar-carbon-coated copper grids. The grids were then incubated in 1% glutaraldehyde for 5 min, washed with deionized water eight times, and then negatively stained with 2% uranyl oxalate (pH 7) for 5 min and methyl cellulose/uranyl for 10 min at 4 °C. Excess methyl cellulose/uranyl was blotted off, and the grids were air-dried and observed with a TEM (Morgagni 268D, Philips Electronics, Eindhoven, The Netherlands)) at an accelerating voltage of 80 kV. Digital images were taken with Mega View imaging software. DLS experiments were performed to measure MVs size as previously described [60]. 

For flow cytometry analysis, about 5 μg of MVs were labeled with different antibodies CD45, CD90, CD73, CD105 or specific isotypes. The size of MVs was estimated by comparing the forward scatter signals with those of reference microspheres obtained from flow cytometry sub-micron particle size reference kit (Thermo Fisher Scientific). Samples were acquired using a FACSCanto (BD Biosciences) and data analysis was performed using the FlowJo program. 

### 4.12. Statistical Analysis

Statistical significance between two groups was determined by performing two-tailed, paired Student’s *t*-test. Differences between multiple groups were analysed with two-way analysis of variance (ANOVA). GraphPad Prism 6 (GraphPad, San Diego, CA, USA) software was used. Graphs show mean values, and error bars represent the SD or SEM. 

## 5. Conclusions

In this study, we provide evidence of a novel mechanism controlling PVR expression on MM cells. We demonstrate that BMSCs enhance PVR surface expression on MM cells and promote their NK cell-mediated recognition. PVR upregulation occurs at transcriptional level and involves NF-kB transcription factor activation by BMSC-derived soluble factors. IL-8-bearing MVs released by BMSCs are essential for PVR upregulation and emerged as novel important regulators of this ligand. The newly uncovered role for BMSCs and IL-8 in supporting the expression of this molecule on MM cells will be useful for therapeutic applications aimed at enhancing the immune-mediated attack of these cancer cells.

## Figures and Tables

**Table 1:** Clinical parameters of MM patients used for the analysis of PVR surface expression and mRNA. Patients were classified according to Durie and Salmon’s Staging System.

Patient	Sex/Age	Clinical Stage	Monoclonal Ig	% of PCs
1	M/74	MM PD	IgG-ʎ	8
2	F/63	Relapse	IgGλ	90
3	M/65	Relapse	IgG-ʎ	70
4	M/61	Relapse	IgG-k	55
5	M/67	Relapse	IgG-k	15
6	M/73	Onset	IgG-ʎ	61
7	F/74	Onset	IgG-ʎ	23
8	M/65	Relapse	IgG-ʎ	29
9	F/74	Smoldering	IgG-k	64
10	F/70	Onset	IgG-k	48
11	M/79	Relapse	IgG-k	34
12	M/59	Smoldering	IgGk	40
13	M/55	Onset	IgG-k	38
14	M/61	Relapse	IgG-k	57
15	F/57	Smoldering	IgG-ʎ	10
16	F/75	MGUS	IgG-ʎ	7
17	M/80	Onset	IgG-k	25
18	M/88	Onset	IgG-k	15
19	F/60	Onset	IgG-ʎ	25
20	F/71	Smoldering	IgG-k	4

**Table 2:** Clinical parameters of MM patients used for the analysis of IL-8 production by BMSCs. Patients were classified according to Durie and Salmon’s Staging System.

Patient	Sex/Age	Clinical Stage	Monoclonal Ig	% of PCs
1	M/74	MM PD	IgG-ʎ	8
2	F/63	Relapse	IgGλ	90
3	M/65	Relapse	IgG-ʎ	70
4	F/76	Onset	IgG-k	55
5	M/67	Relapse	IgG-k	15
6	M/61	Onset	IgG-ʎ	61
7	F/74	Onset	IgG-ʎ	23
8	M/73	Relapse	IgG-ʎ	29
9	F/74	Smoldering	IgG-ʎ	64
10	F/70	Onset	IgG-k	48
11	F/62	Onset	IgG-k	48
12	M/59	Smoldering	IgG-k	69
13	M/68	Relapse	IgG-k	9
14	F/65	Onset	IgG-ʎ	73
15	F/67	Onset	IgG-ʎ	22
16	M/79	Relapse	IgG-k	34
17	M/55	Onset	IgG-k	38
18	M/61	Relapse	IgG-k	57

## Supplementary Materials

The following are available online at https://www.mdpi.com/2072-6694/12/2/440/s1, Figure S1: Phenotypic characterization of human MM patient-derived BMSCs, Figure S2: Effects of BMSC-CM on MM cell vitality, Figure S3: PVR expression correlates with CXCR1 and CXCR2 expression in MM patients, Figure S4: Characterization of BMSC-derived microvesicles, Figure S5: BMSC-CM-derived proteins >100 KDa induce PVR expression on MM cells, Figure S6: Western blotting: complete gels reported in Figure 6.
