# Supplementary material for: Bone Marrow Stromal Cell-Derived IL-8 Upregulates PVR Expression on Multiple Myeloma Cells via NF-kB Transcription Factor
Source: Cancers (Basel). 2020 Feb 13;12(2):440. doi: 10.3390/cancers12020440 (PMC7072437; doi:10.3390/cancers12020440)
Supplement: Supplementary file 1 [file cancers-12-00440-s001.pdf]

cancers

MDPI

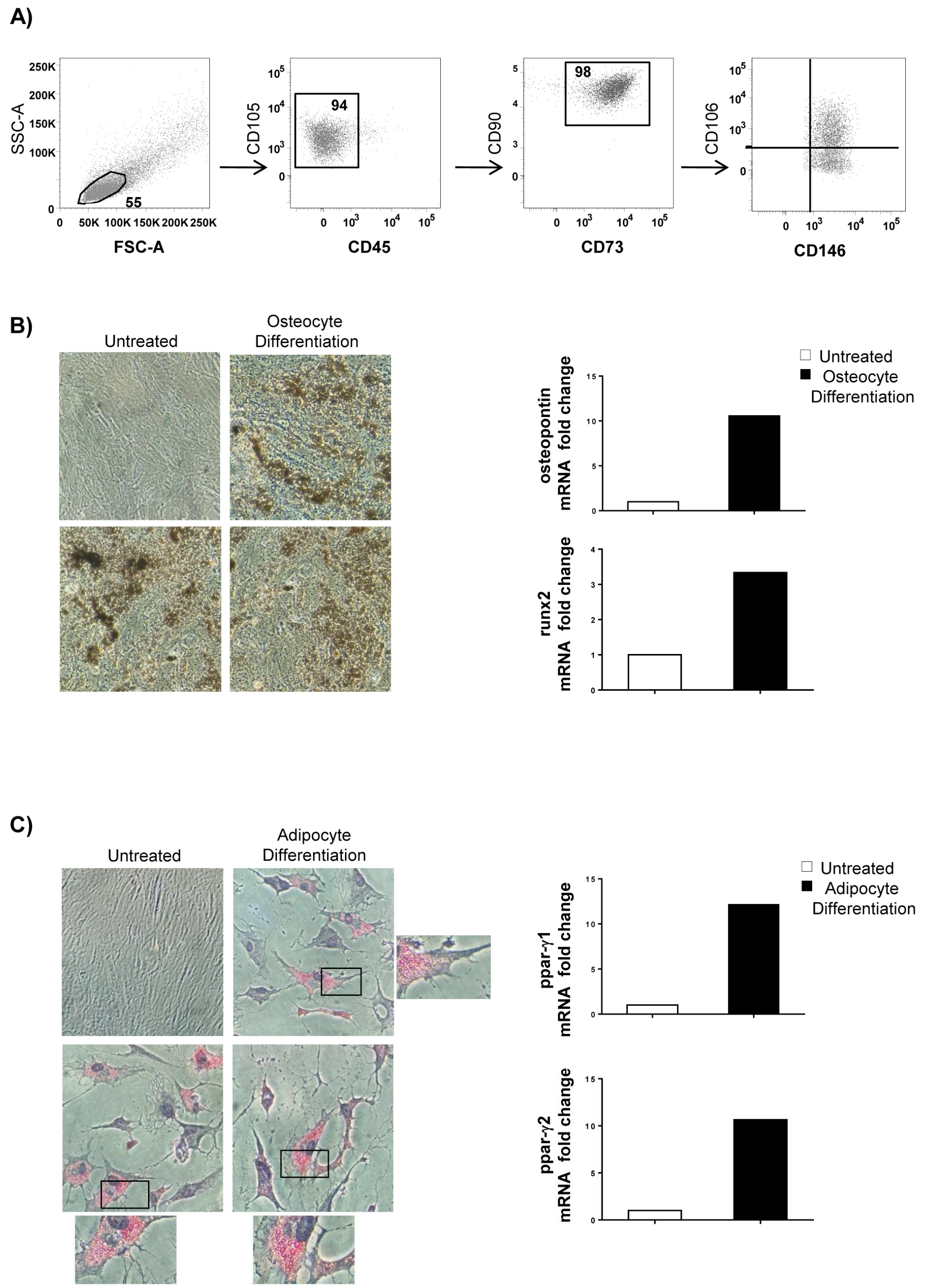

Figure S1. Phenotypic characterization of human MM patient-derived BMSCs: (**A**) Primary BMSCs isolated from CD138 (−) fraction of BM mononuclear cells from Active MM patients were subjected to multiparametric cytofluorimetric analysis. Dot plots represent an example of gating strategy used. BMSCs were growth in osteogenic (**B, left panel**) or adipogenic medium (**C, left panel**) and assayed by von Kossa or Red Oil staining to evaluate their capacity to generate osteocytes or adipocytes. Total RNA was isolated from differentiated cells and assayed for the expression of osteogenic (Osteopontin and Runx2) (**B, right panel**) or adipogenic genes (PPAR-γ1 and PPAR-γ2) (**C, right panel**) by Real Time PCR analysis. Data, expressed as fold change units, were normalized with GAPDH and referred to the untreated cells, considered as calibrator. One out of three independent experiments is shown.

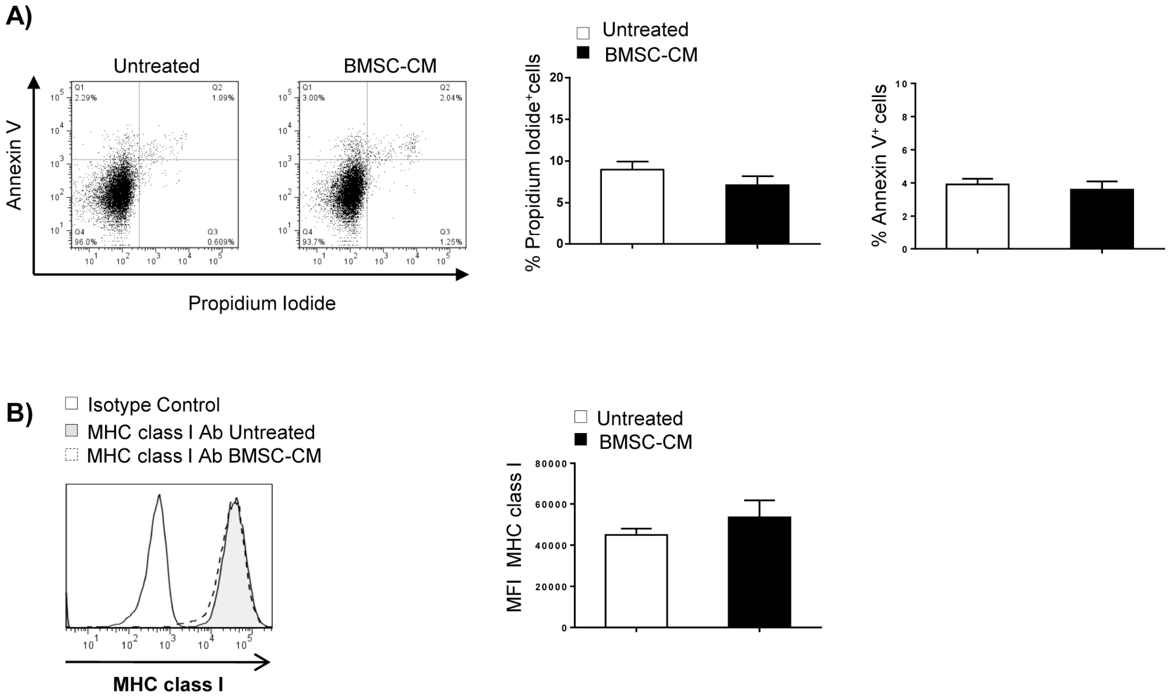

Figure S2. Effects of BMSC-CM on MM cell vitality. (**A**) SKO-007(J3) untreated or treated with BMSC-CM for 72 h were stained using Annexin-V/APC and Propidium Iodide. A representative experiment is shown (left panel). Histograms indicate the percentage of Annexin V or propidium positive cells and were calculated based on at least 3 independent experiments (right panels). (**B**) MHC class I expression was analysed by flow cytometry on SKO-007(J3) cells cultured for 72h in BMSC-CM. Histograms represent the MFI of specific mAb - MFI of isotype control. The MFI of MHC class I was calculated based on at least three independent experiments.

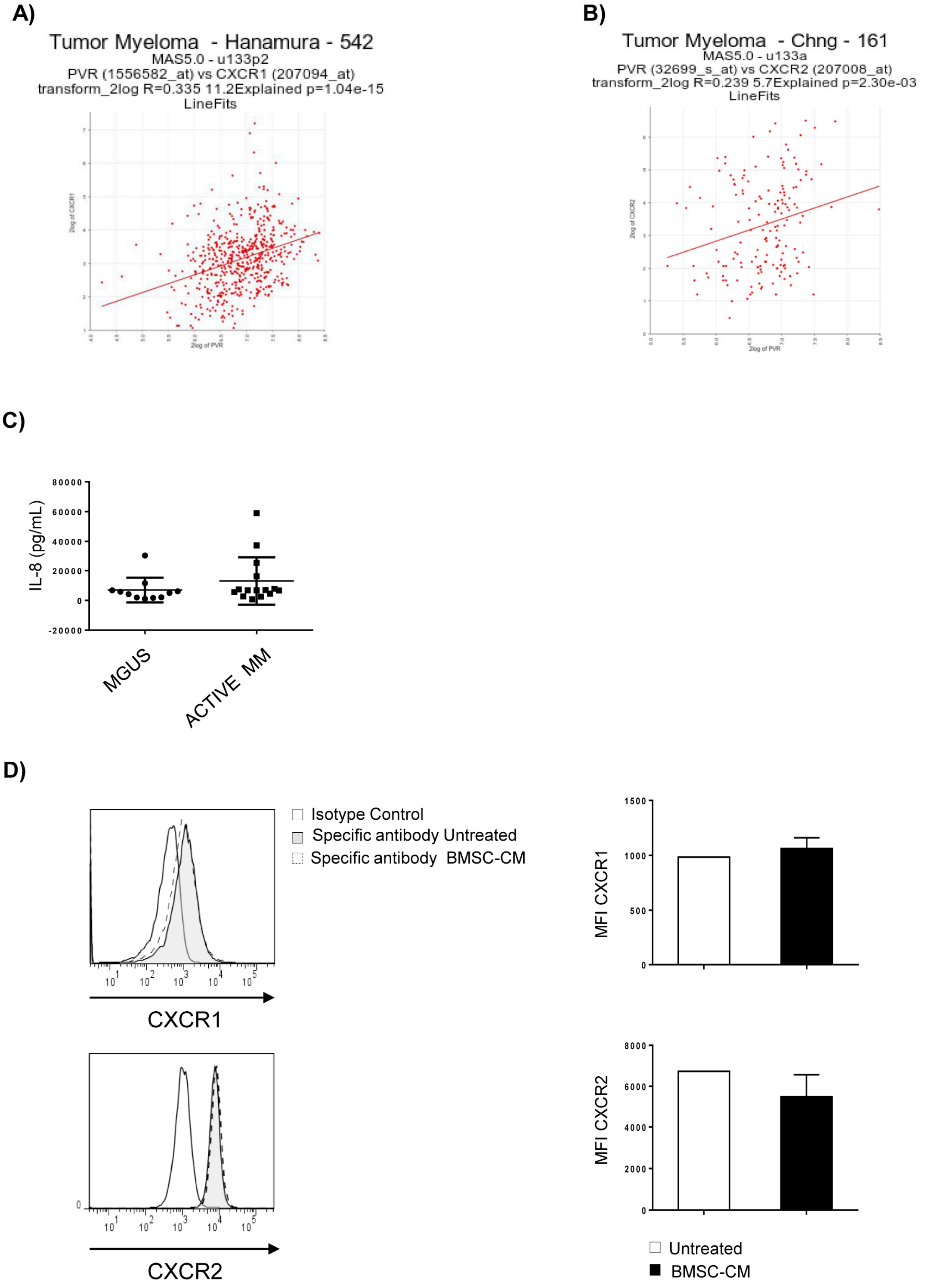

Figure S3. PVR expression correlates with CXCR1 and CXCR2 expression in MM patients. (**A**) Correlation analysis of expression values from MM patients between PVR and CXCR1 (probe set 1556582_at for PVR vs. probe set 207094_at for CXCR1). R-value: 0.335, *p*-value = 1.04e^{-15}, (Hanamura MM Dataset of R2), and (**B**) between PVR and CXCR2 (probe set 32699_at for PVR vs. probe set 207008_at for CXCR2). R-value: 0.239, *p*-value = 2.30e^{-03}, (Chng-161 MM Dataset of R2). BMSCs secrete IL-8 and MM cells express CXCR1 and CXCR2 receptors. (**C**) MGUS or Active MM-BMSCs were cultured in serum-free MEMα medium for 72h and then conditioned medium was assayed for IL-8 by ELISA. (**D**) CXCR1 and CXCR2 expression was analysed by flow cytometry on SKO-007(J3) cells cultured for 72h in BMSC-CM. Histograms represent the MFI of specific mAb - MFI of isotype control, which was calculated based on at least three independent experiments.

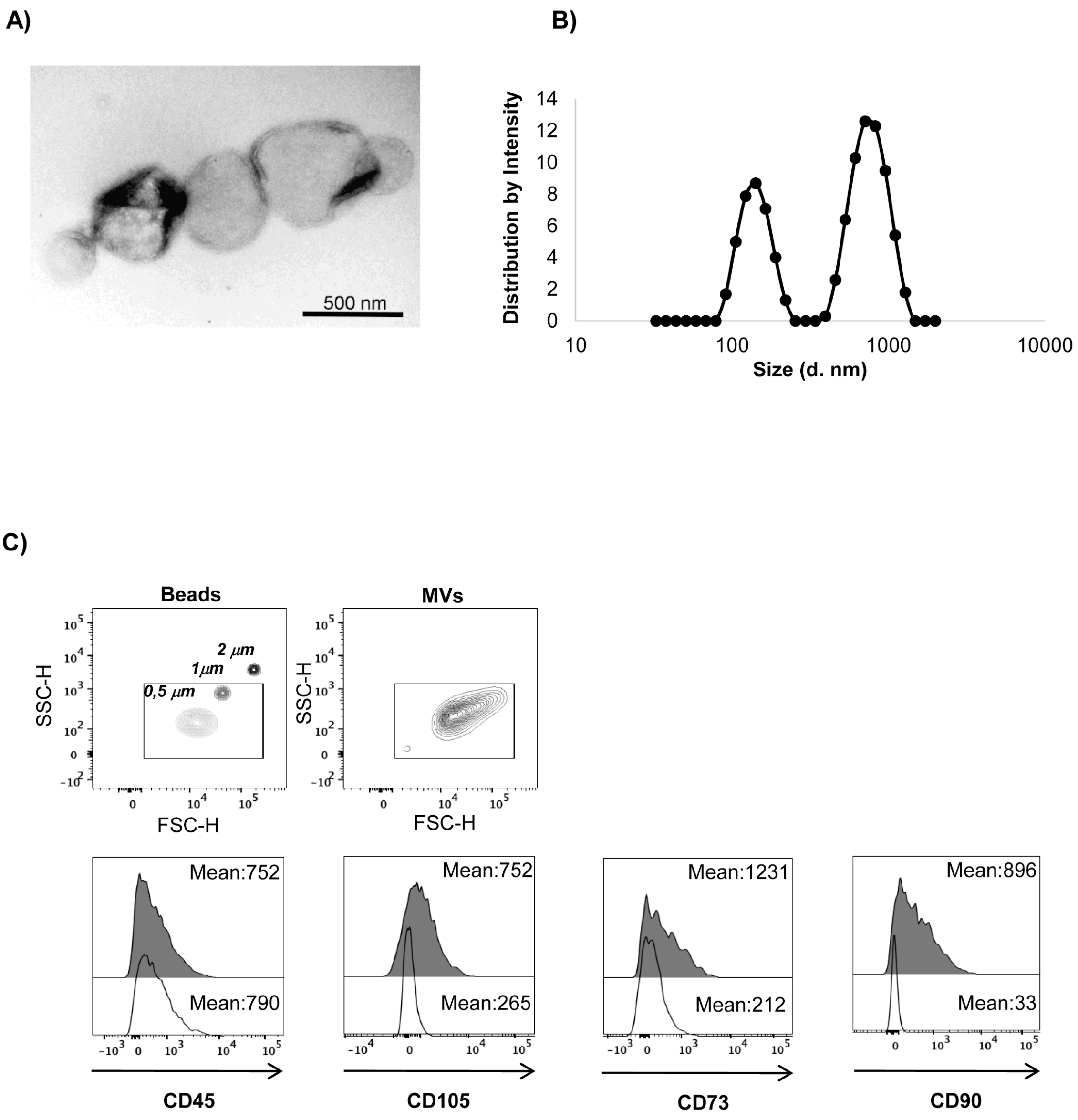

Figure S4. Characterization of BMSC-derived microvesicles. (**A**) Electron microscope analysis of microvesicles (MVs) morphology and size. A representative image of MVs prepared from BMSCs is shown. (**B**) Size distribution of BMSC-derived MVs was analysed through DLS. A representative experiment out of three is shown. (**C**) Flow cytometry characterization of MVs released by BMSCs. Reference size beads were used to gate MVs, defined as elements of smaller size than 1μm (upper panel). The grey colored histograms represent MVs stained with negative (CD45) and positive (CD105, CD73, and CD90) BMSC markers, while white histograms represent isotype control (lower panel).

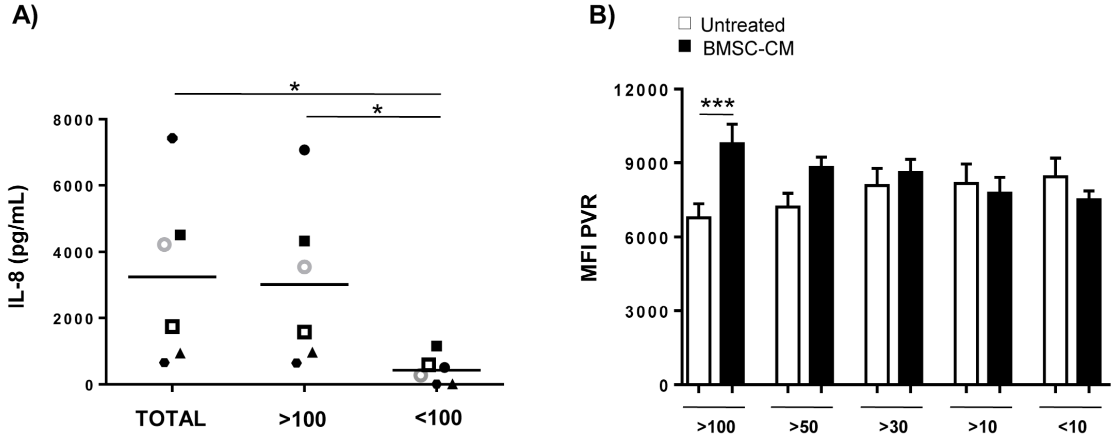

Figure S5. BMSC-CM-derived proteins >100KDa induce PVR expression on MM cells. (**A**) Total and fractioned (<100 or >100 KDa) BMSC-CM (**D**) were analysed for IL-8 by ELISA. Data were calculated based on at least 3 independent experiments ± SD (*p< 0.05; ANOVA). (**B**) PVR surface expression was analysed by flow cytometry on SKO-007(J3) cells cultured for 72h in fractions of different molecular weight obtained from BMSC-CM or complete RPMI1640 medium. Histograms represent the MFI of specific mAb - MFI of isotype control, which was calculated based on at least three independent experiments ± SEM (*p < 0.05; **p < 0.005; ***p < 0.002; ****p < 0.001; paired Student t-test).

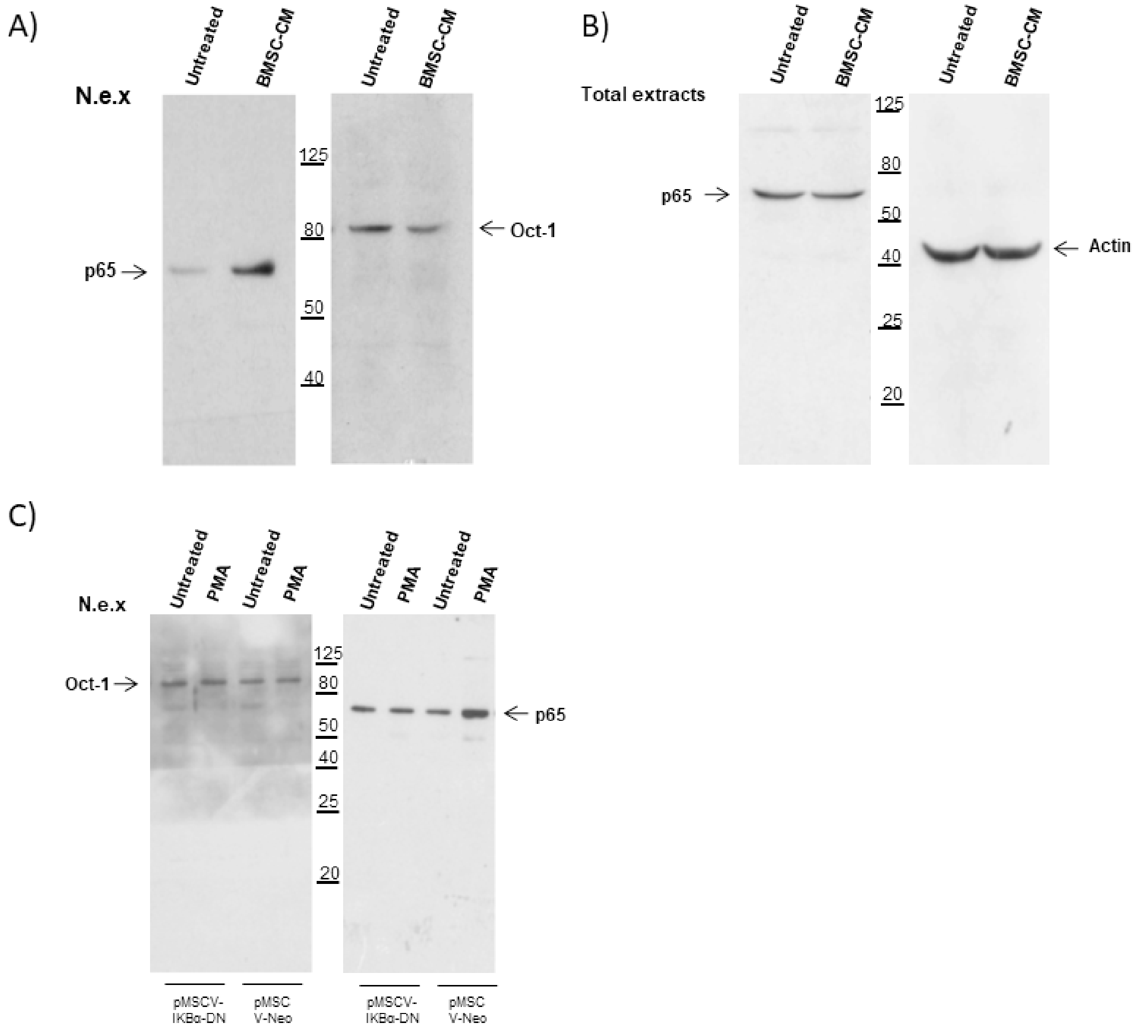

Figure S6. Western blotting: complete gels reported in Figure 6.
